# Application and development of hydrogel biomaterials for the treatment of intervertebral disc degeneration: a literature review

**DOI:** 10.3389/fcell.2023.1286223

**Published:** 2023-12-07

**Authors:** Yuheng Liu, Zhen Zhao, Chuan Guo, Zhangheng Huang, Weifei Zhang, Fei Ma, Zhe Wang, Qingquan Kong, Yu Wang

**Affiliations:** Department of Orthopedic Surgery and Orthopedic Research Institute, West China Hospital, Sichuan University, Chengdu, Sichuan, China

**Keywords:** hydrogel, intervertebral disc, degeneration, regeneration, stimulus response

## Abstract

Low back pain caused by disc herniation and spinal stenosis imposes an enormous medical burden on society due to its high prevalence and refractory nature. This is mainly due to the long-term inflammation and degradation of the extracellular matrix in the process of intervertebral disc degeneration (IVDD), which manifests as loss of water in the nucleus pulposus (NP) and the formation of fibrous disc fissures. Biomaterial repair strategies involving hydrogels play an important role in the treatment of intervertebral disc degeneration. Excellent biocompatibility, tunable mechanical properties, easy modification, injectability, and the ability to encapsulate drugs, cells, genes, etc. make hydrogels good candidates as scaffolds and cell/drug carriers for treating NP degeneration and other aspects of IVDD. This review first briefly describes the anatomy, pathology, and current treatments of IVDD, and then introduces different types of hydrogels and addresses “smart hydrogels”. Finally, we discuss the feasibility and prospects of using hydrogels to treat IVDD.

## 1 Introduction

The intervertebral disc (IVD) is a connective structure located between two adjacent vertebrae of the spine, consisting of the nucleus pulposus (NP), the annulus fibrosus (AF), and the cartilaginous endplate (CEP), and its main function is to provide flexibility and stability for the spine (Shapiro et al., 2012). Due to its location, the IVD is also an important load-bearing organ.

The IVD is the largest avascular organ in the human body, with only a small amount of vascularization present in the cartilaginous endplates (CEPs) and the outer lamellae of the AF (Dowdell et al., 2017; Bermudez-Lekerika et al., 2022). Almost all exchanges of nutrients and waste products occur through CEP vascular infiltration and extracellular matrix diffusion (Fields et al., 2018; Kim et al., 2022). With age, degenerative changes such as ossification of the cartilage endplate decrease its permeability and blood supply, which exacerbates the hypoxic environment inside the IVD, mainly manifested by lactic acid accumulation and a decrease in ambient pH. These environmental factors accelerate cell death (Choi et al., 2018; Silagi et al., 2020). These age-related risk factors cumulatively lead to a greater susceptibility of the IVD to degenerative disease.

IVDD often causes low back pain as well as neck and upper back pain, accompanied by numbness of the upper and lower extremities (Wen et al., 2022). In severe cases, weakness of the limbs, and nerve damage symptoms can occur. Over time, these symptoms lead to a large number of patients with disabilities, of which low back pain is one of the most important public problems, and has a major impact on public health, resulting in a serious socioeconomic burden. As the population ages, low back pain and other diseases caused by intervertebral discs are receiving increasing attention (Samanta et al., 2023).

The intervertebral disc belongs to the fibrocartilage part of the “three-joint complex” (Oichi et al., 2020), which connects two adjacent vertebrae, maintains pressure, and provides flexibility for spinal activities. It also forms an anatomical structure, in combination with the upper and lower vertebral bodies, that protects the spinal cord nerves. As the intervertebral disc ages and the pressure on the intervertebral disc increases, it leads to herniation of the intervertebral disc and narrowing of the spinal canal, which causes the symptoms mentioned above.

The main factors causing intervertebral disc degeneration include the destruction of the extracellular matrix (ECM), changes in IVD cell phenotypes, and the progressive inflammatory response in the IVD (Rider et al., 2019). At present, the commonly used conservative treatment are nonsteroidal anti-inflammatory drugs and analgesics, however the vast majority of patients need surgery to relieve symptoms, although the effect of surgery on patients with multisegment degeneration is not satisfactory (Xin et al., 2022). In addition, the recurrence rate after surgery is high, leading to problems such as degeneration of the adjacent segment IVD. Therefore, conventional conservative treatment and surgical treatment can be considered palliative treatments, which can relieve symptoms but not reverse the degeneration of the intervertebral disc and restore its function (Raj, 2008; Vasiliadis et al., 2014; van Uden et al., 2017; Xin et al., 2022). In recent years, cell therapy, drug therapy and gene therapy have been gradually used to treat degenerative intervertebral discs, and mesenchymal stem cells (MSCs) (Hu et al., 2020; Cunha et al., 2021) and platelet-rich plasma (PRP) (Nagae et al., 2007; Chai et al., 2023) have even entered clinical trials. However the delivery of these therapeutic factors to the intervertebral disc has not been widely put into clinical use due to the lack of high-quality clinical evidence (Kawabata et al., 2023). Prolonged inflammation within a degenerating disc can lead to a hostile ECM environment in which drugs and other bioactive agents are readily degraded. Therefore, it is important to find a material that can carry biological agents, protect them from *in-situ* degradation, and enable long-term release. Hydrogels are advantageous due to their high hydration properties (Ma et al., 2019; Zhang et al., 2021).

Materials used for intervertebral disc treatment should have high strength, high flexibility and high toughness (Wang et al., 2023a). Polymer hydrogels have a three-dimensional cross-linked network structure and adjustable physical and chemical properties (biocompatibility, biodegradability, material transport, and mechanical strength), which are conducive to cell adhesion and proliferation; therefore, they have been widely studied in relation to IVDD (Correa et al., 2021). Hydrogels can simulate the structure and mechanical properties of human tissue by changing the type, molecular weight, cross-linking degree, and other properties of the material, and can further load cells, drugs, growth factors, etc., thereby delivering cellular nutrients and releasing active drugs to promote the regeneration of degenerative NP (Ahmed, 2015; Correa et al., 2021). Physical or chemical cross-linking methods can also be used to adjust the swelling rate, degradation rate, biocompatibility and other properties of hydrogels to treat the corresponding diseases (Correa et al., 2021). Therefore, this article is intended to summarize the advantages of hydrogel materials, introduce various types of hydrogels, analyze the modification of different hydrogels to overcome existing limitations and discuss their clinical application prospects for IVDD treatment.

## 2 Degeneration of IVD

### 2.1 Biomechanical causes

**FIGURE 1:**
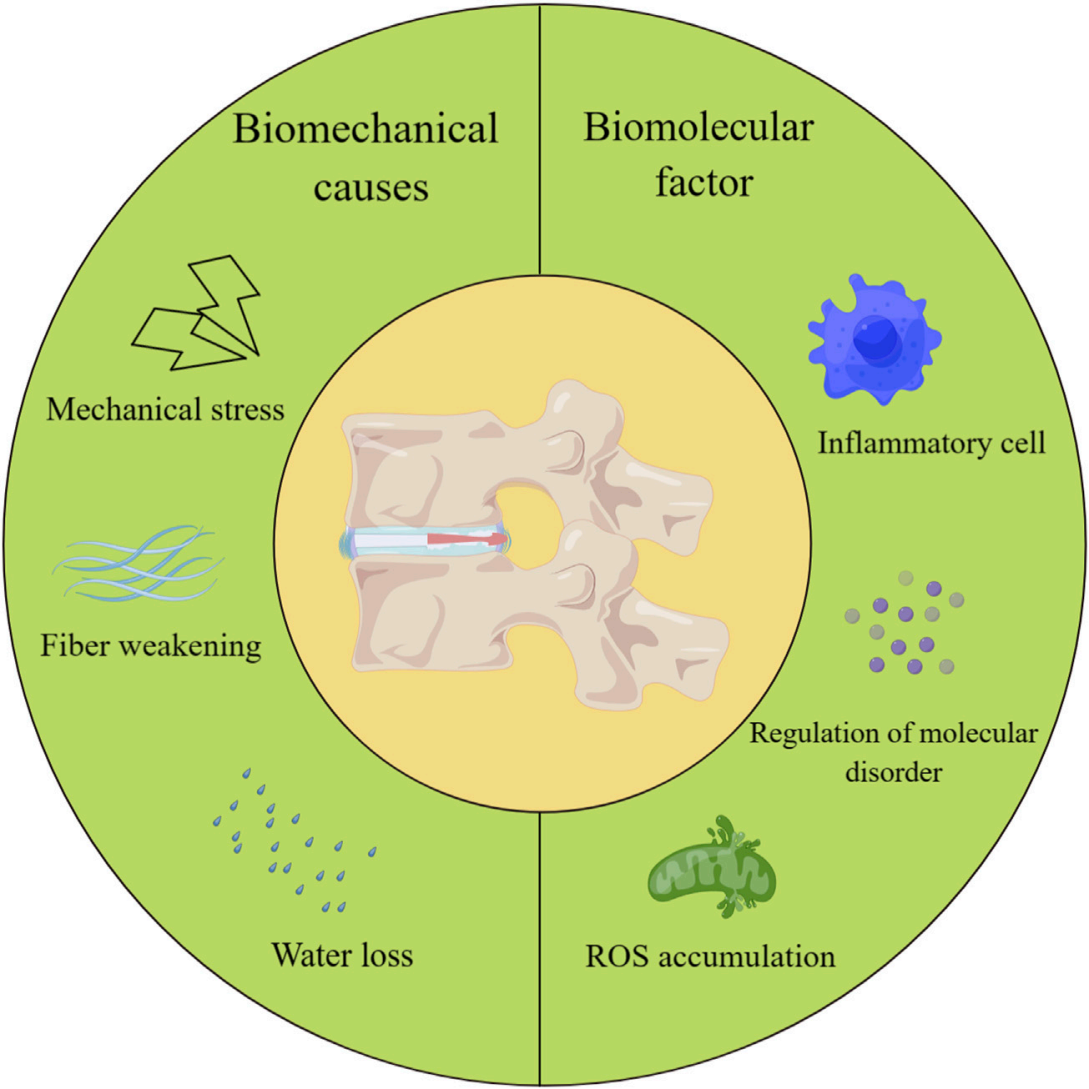
Biomechanical causes and biomolecular factors leading to IVDD.

IVDD is a common spinal disorder that occurs mainly after long-term mechanical stress and changes in biological environmental factors. Due to the physiological and anatomical position of the IVD, stress transmission is one of its main functions. Under normal circumstances, the IVD functions effectively in support and cushioning. However, when an organism is affected by various factors, the structure and function of the IVD tissue may undergo degeneration and injury (Figure 1) (Buckwalter, 1995; Urban and Roberts, 2003; Adams and Roughley, 2006; Sakai et al., 2012).

Many studies have demonstrated that age is one of the main risk factors for IVDD (Wang et al., 2016; Ohnishi et al., 2018; Li et al., 2022). With age, the water content of the IVD gradually decreases, and the water content of the annulus fibrosus and nucleus pulposus decreases, reducing the elasticity and flexibility of the IVD (Rasekhi et al., 2006). In addition, as we age, the body produces less collagen and other important components, weakening the regenerative capacity of the IVD (Pokharna and Phillips, 1998; Roberts et al., 2006).

Poor lifestyle habits are also among the most important factors leading to IVDD. For example, maintaining the same position for long periods of time and incorrect posture can increase the load on the IVD, causing excessive stress and injury (Williams and Sambrook, 2011). Prolonged sitting, sedentary behavior and lack of exercise can lead to poor blood circulation in the discs and accelerate the degeneration process (Ding et al., 2021).

In addition, trauma and injury can cause degeneration. For example, sudden heavy pressure, sprains, or vibration in a long-term work environment can cause damage. Such injuries can dehydrate IVD tissue (Figure 1).

### 2.2 Biomolecular factors

During the onset of IVDD, the first thing that occurs is an imbalance in the synthesis and degradation of the extracellular matrix (Kirnaz et al., 2022; Mohd Isa et al., 2022). This changes the matrix composition of the IVD tissue. This may involve the uncoordinated action of a variety of regulatory molecules such as cytokines, growth factors, and degradative enzymes such as matrix metalloproteinases (MMPs) and a disintegrin and metalloproteinase with thrombospondin motifs (ADAMTs) (Le Maitre et al., 2006; Rutges et al., 2008; Zhao et al., 2011; Wang et al., 2013; Zhao and Li, 2021). This leads to a disruption of extracellular matrix catabolism and synthetic homeostasis (Figure 1).

On the other hand, degeneration of the IVD is also associated with sterile inflammation and oxidative stress. As the IVD is in a hypoxic environment, this results in metabolites not being excreted efficiently (Bartels et al., 1998; Wang et al., 2023b). The accumulation of harmful substances leads to inflammation, which affects cellular function. Oxidative stress is the result of an imbalance in redox dynamics, manifested by an increase in the intracellular levels of reactive oxygen species (ROS) and a relative decrease in the levels of antioxidants, which may compromise the integrity of cellular function (Cheng F. et al., 2022; Wang D. K. et al., 2022; Zhang C. Y. et al., 2022; Cao et al., 2022; Wei et al., 2022; Wen et al., 2022). It has also been shown that the accumulation of ROS is positively correlated with the degree of IVDD (Bai et al., 2020). During this process, IVD cell death is accelerated (Figure 1).

IVDD is also influenced by genetic factors (Siemionow et al., 2011; Williams et al., 2011; Mei et al., 2022). Multiple genes have been shown to correlate with disc degeneration, such as miR-21 (Wang Y. et al., 2022; Wang et al., 2023a; Wang et al., 2023c) and COL9A2 (Xu et al., 2022). With advances in cellular molecular research, gene therapy for IVDs is gradually being developed. The application of RNA interference (RNAi) and CRISPR gene editing technologies has enabled a promising start for gene-targeted therapy for IVDD (Roh et al., 2021).

## 3 Hydrogel for the treatment of IVDD

**FIGURE 2:**
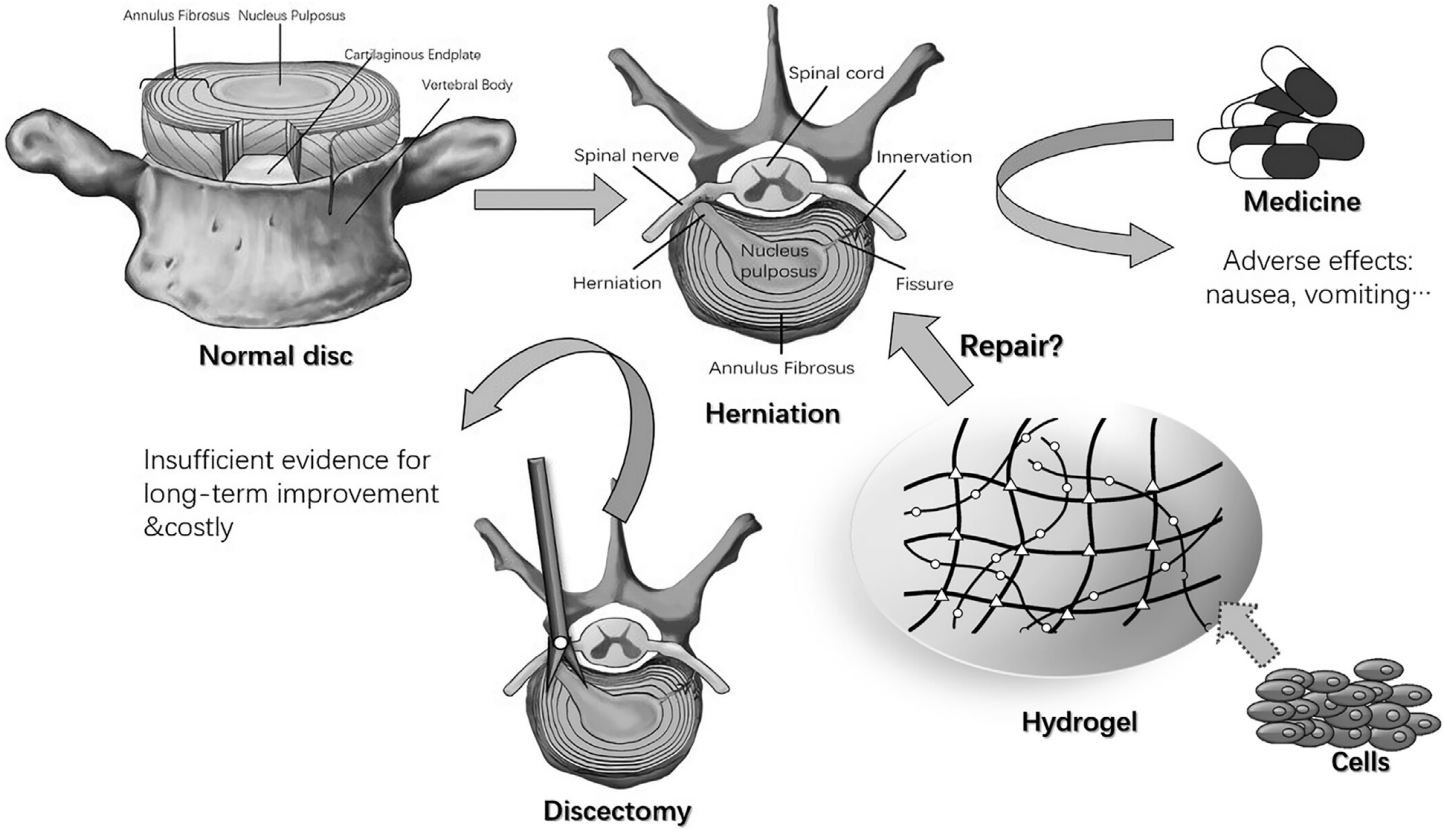
Normal intervertebral disc, herniation, and treatments (Zheng and Du, 2021).

Hydrogels are polymers with a three-dimensional mesh structure formed by cross-linking chains of hydrophilic macromolecules (Ahmed, 2015; Yan et al., 2021; Ho et al., 2022). As injectable biomaterials, hydrogels have strong therapeutic applications and have gained widespread attention in the study of IVDD (Figure 2). First, most hydrogels are biocompatible with biological tissues and do not cause significant immune reactions (Kharaziha et al., 2021; Bu et al., 2022). On the other hand, due to the high water content, hydrogel can provide a favorable moisture environment, which is conducive to the growth and activity of IVD cells (Francisco et al., 2014; Verhulsel et al., 2014; Sun Y. et al., 2016). The physical and chemical properties of hydrogels can be adjusted by adapting formulations and preparation methods to meet different application requirements (Billiet et al., 2012; Zhai et al., 2013; Li et al., 2015; Yu et al., 2015). Therefore, the structure and properties of hydrogels can be regulated by controlling the degree of cross-linking, pore structure and other parameters to achieve the desired functions and properties. In addition, bioactive substances (e.g., cytokines, drugs, etc.) can be added to hydrogels to exert biological functions, such as promoting tissue repair and regeneration (Chai et al., 2017; Kamoun et al., 2017). As the IVD is an avascular enclosed organ, hydrogels that are applied in IVDD treatment must be injectable, to allow minimally invasive implantation. As bioactive therapeutic factor delivery vectors, hydrogels must be biodegradable and microenvironment-responsive (Wang Y. et al., 2022; Wang et al., 2023a; Wang et al., 2023c). All these requirements should be taken into consideration in the design of hydrogels for use in IVDD treatment. However, studies have revealed a number of potential problems that limit the application of hydrogels in IVDD treatment. Hydrogel injection may also cause mechanical damage to the IVD. More minimally invasive injections are a topic of intense interest for future research, and will requires hydrogels with a good ability to change from liquid to solid (Kaur and Murphy, 2023). This property is very favorable for the repair of IVDs, but, it also presents greater difficulty in cross-linking. A hydrogel that is poorly designed for cross-linking has low mechanical strength and susceptibility to deformation and rupture, limiting its use in certain applications (Baumgartner et al., 2021). Moreover, hydrogels have a certain water solubility, which is easily affected by ambient moisture, limiting their application in certain moist environments. Water solubility also limits the stability of hydrogels, which are easily affected by temperature, pH and other factors and need to be modified and protected appropriately (Woodard and Grunlan, 2018). To solve the abovementioned problems of injectable hydrogels, a variety of dynamically cross-linked hydrogels are being vigorously pursued. Next, we will list some commonly used hydrogel matrix materials and the application of intelligent environment-responsive hydrogels in IVDD repair.

### 3.1 Skeleton materials for hydrogels

We have summarized the crosslinking mechanism as well as the advantages and disadvantages of hydrogels designed with different materials (both natural and synthetic) in recent years in Table 1.

**TABLE 1:** Cross-linking mechanisms and advantages and disadvantages of hydrogels designed in recent years using different substances.

Biomaterial components	Year	Crosslinking mechanism	Main advantage	Main shortage	Reference
Chitosan/alginate	2023	polyelectrolyte complexation	High water content, controlled mechanical properties, excellent biocompatibility	Stent system instability	Ye et al. (2023)
Gelatin	2023	glutaraldehyde cross-linking	Low immunogenicity, integrins can be introduced	Unstated	Yahia et al. (2023)
Glass/sodium	2023	calcium ion cross-linking	High drug delivery efficiency, low degradation products, and it takes only a few minutes at room temperature to achieve complete curing	Potential cytotoxicity	Wu et al. (2023)
Fibrinogen	2023	Genipin cross-linking	Good anti-inflammatory properties, highly biocompatible, it can be made and repaired precisely	Unable to withstand the daily pressure load of human intervertebral discs, practical application in the clinic becomes difficult	Wang et al. (2023e)
PEG/miRNA	2023	amino-yne click reaction	This hydrogel has excellent mechanical strength, high plasticity, structural stability, and sustainable drug release	Potential effects of miRNAs on target genes	Wang et al. (2023a)
Poly(ethyleneglycol)/decellularized cell-derived matrix (dNCM)	2023	PEG-diurethane-dithiol crosslinker	The hydrogel can assume a stiffness close to the intended target tissue within the disc, aiding biomechanical repair of the joint in a non-swollen state	Potential impact on IVD cells *in situ*	Schmitz et al. (2023)
Gelatin	2023	bioorthogonal	Provides payload, reduces pain and promotes histological recovery	Unstated	Luo et al. (2023a)
Chitosan/PEG	2023	Schiff base reaction/photo-crosslink	Delayed disc degeneration, biocompatible	Difficulty in combining rapid gelation and increased physical stresses	Huang et al. (2023b)
Hyaluronate	2023	Diels–Alder reaction	This hydrogel exhibits favorable injectability, mechanical property, good antioxidant properties, pH-responsive delivery behavior, and inhibits inflammatory factors	Mechanical properties still need more clinical testing	Luo et al. (2023b)
Gelatin	2022	Schiff’s base reaction	Good antimicrobial, antioxidant and mechanical properties. Minimally invasive injections are possible	Unstated	Yang et al. (2022b)
Peptide	2022	self-assembly	High water content, injectable line, good biocompatibility, degradability	Potential effects of cellular autophagy have not been ruled out	Tang et al. (2022)
Poly (vinyl alcohol)	2022	glycerol cross-linking	Reliable biosafety with gel properties similar to natural medulla oblongata, highly injectable	The feasibility of clinical application requires further follow-up studies	Jia et al. (2022b)
Alginate	2022	calcium ion cross-linking	Better mechanical stability, excellent water-absorbent properties, promote cell repair and regeneration	Unstated	Li et al. (2023d)
Hyaluronate	2022	unstated	Good physical properties and biocompatibility at the same time	Tests use rat models with large gaps in human tissue, more research needed for clinical application	Hu et al. (2022)
Poly(vinyl alcohol)	2022	unstated	Low toxicity, good biocompatibility, high mechanical strength	More research is still needed on materials with different weight percentages	Heo and Park (2022)
polyethylene glycol fumarate/sodium methacrylate (OPF/SMA)	2022	situ formed	Continuous anti-inflammatory repair and protection of the intervertebral disc	Failure to fully simulate the microenvironment of intervertebral disc degeneration in the test	Cheng et al. (2022b)
extracellular matrix	2021	situ formed	Biocompatible, excellent small molecule drug delivery, ease of access to raw materials	ECM enrichment and material preparation is difficult	Xing et al. (2021)
Hyaluronan/poly(N-isopropylacrylamide)	2021	situ formed	Induction of endogenous cell recruitment; Recruitment of MSCs for vein transplantation; Promotion of IVD regeneration in IVD lesion patterns	Further research is needed on the type of cells to be recruited and there is a potential effect on the time of measurement of the experimental metrics	Cunha et al. (2021)
PEG/peptide	2021	situ formed	Excellent bioactivity and substrate synthesis promoting function	Inability to provide sufficient mechanical stress and difficulties with hydrogel injection	Barcellona et al. (2021)
Gelatin	2021	situ formed	Good cytocompatibility, excellent stability, mechanical properties, minimally invasive injections possible	Poor nutrient availability in anoxic microenvironments	Wang et al. (2021)
hyaluronic acid (HA)/platelet-rich plasma (PRP)	2021	Batroxobin(BTX)	Promotes tissue repair and can provide an effective microenvironment for stem cells	No simulation of effects on tissue cells under biomechanical stimulation	Russo et al. (2021)

#### 3.1.1 Natural polymers

There are many types of natural polymers that can be used as materials for hydrogels. Common natural polymers include proteins, polysaccharides, and peptides. These natural polymers can usually be extracted from naturally occurring substances (Chuah et al., 2017). These materials have desirable biocompatibility and biodegradability, but they also have certain defects, such as lacking the needed mechanical properties.

##### 3.1.1.1 Chitosan

Chitosan is the product of N-deacetylation of chitin. It is often found in the shells of marine arthropods (Li et al., 2017). It is the second largest natural macromolecule and has various advantages characteristics such as biocompatibility, biodegradability, bacteriostasis, and nontoxicity, which has led to its widespread use in many fields, such as medical equipment and tissue engineering carrier materials (Bhattarai et al., 2010). Chitosan has excellent hydrophilicity and water absorption capacity because it contains a large number of hydroxyl groups and amino groups. Some studies have shown that adding β-glycerophosphate (BGP) to chitosan can cause it to gelate at normal physiological temperature (Chenite et al., 2000); based on this feature, chitosan can be injected into the desired area for *in-situ* gelation, but it has poor mechanical properties and long gelation time. To solve the problems of pure chitosan gels, chitosan is usually used as a material in composite hydrogels, such as chitosan, gelatin, glutaraldehyde and other materials to form a new hydrogel system. To achieve the needed mechanical strength and prepare pH-sensitive (Van der Schueren et al., 2013) or temperature-sensitive (Liu S. et al., 2021) hydrogels, chitosan can also be physically cross-linked with polyvinyl alcohol to form a conductive hydrogel system (Zhang et al., 2023). The recent literature has reported an injectable chitosan/PEG hydrogel, that rapidly undergoes gelation *in situ* by a Schiff base reaction, further enhanced by blue light irradiation, and can effectively promote the proliferation of NP cells(NPCs) for the repair of degenerative IVDs (Huang et al., 2023a).

##### 3.1.1.2 Cellulose

Cellulose is the largest natural polymer and has good biocompatibility and adjustable mechanical properties. Its derivatives include carboxymethyl cellulose (CMC) (Zheng et al., 2015), methyl cellulose (MC) (Kim et al., 2018a), and hydroxypropyl methyl cellulose (HPMC) (Hu et al., 2018), which have very good biocompatibility, biodegradability and water absorption performance. CMC has a highly negative charge similar to that of the NP. MC can undergo thermal gelation at temperatures above 32°C (Kim et al., 2018b). HPMC has the ability to perform controlled drug release. Injectable CMC-MC polymers can be fabricated by modifying both polymers with methacrylate groups, and the resulting gel can provide adequate mechanical support to restore disc height in injured IVDs (Varma et al., 2018). Cellulose is also often used as a component in composite hydrogels in current research, such as in double network hydrogels with alginate designed to increase the mechanical properties of the hydrogel system (Ho et al., 2023), Cellulose is also used as a component in composite hydrogels to replace the nucleus pulposus in the treatment of IVDD, for example, a poly(ethylene glycol) dimethacrylate nanoprotofibrillated cellulose composite hydrogel was designed by Schmocker et al. to replace the NP tissue (Schmocker et al., 2016). As reported in the latest literature, an alternative scaffold for AF was made from alginate and cellulose with interstitial injection of cellulose solution to mimic the NP, and the resulting scaffold exhibits shape memory and is able to withstand compression and restore disc height. The scaffold carries MSC homing peptide (SKP peptide), cell adhesion peptide (RGD peptide), and GDF-5. SKP peptide recruits MSCs to the injury site, RGD peptide enhances cell attachment, and GDF-5 induces the differentiation of MSCs into NP-like cells and increases Col II and aggrecan synthesis, making this material a promising alternative to disc replacement (Ho et al., 2023).

##### 3.1.1.3 Hyaluronic acid(HA)

HA is the most common material used to make hydrogel systems for IVD applications. HA is a glycosaminoglycan (GAG) that is ubiquitously present in the ECM and on cell surfaces (Mohd Isa et al., 2018; Guo et al., 2022). HA hydrogels have been shown to maintain tissue water content, suppress inflammation, reduce nociceptor expression, and mimic the tissue environment of IVD (Yang et al., 2022a). There are many ways to modify HA. For example, the introduction of thiol groups can result in controllable self-gelation. Hyaluronic acid methacrylate(HAMA) can be cross-linked by light, and the mechanical and biodegradable properties of hydrogel systems can be changed by varying the duration and intensity of light exposure (Eke et al., 2017; Chen et al., 2021). Because of its porous structure and high water content, HAMA can provide a suitable growth environment for NPCs and has promise for application in the treatment of IVDD (Shen et al., 2022). Derek et al. have also used HA and poly(N-isopropylacrylamide) to form a thermoreversible hydrogel, which changes from liquid to gel when the temperature exceeds 30°C (Mortisen et al., 2010). Moreover, since HA acts as a ligand for CD44, a transmembrane glycoprotein involved in various biological functions, a corss-linked HA hydrogel can also enable NPCs to express higher levels of CD44, consequently attenuating cellular inflammation (Isa et al., 2015). J Shen et al. designed an injectable microsphere (MS@MCL) for local lactate exhaustion was constructed by grafting manganese dioxide (MnO_2_)-lactate oxidase (LOX) composite nanozyme on microfluidic hyaluronic acid methacrylate (HAMA) microspheres via chemical bonds. The use of MS@MCL in NPCs culture exhibited excellent ROS clearance, reduced the expression of TNF-α and IL-1β, and increased the expression of Aggrecan and Col II, which effectively inhibited inflammation, promoted the regeneration of ECM, and showed long-term therapeutic effects on IVDD (Shen et al., 2022).

##### 3.1.1.4 Alginate

Alginate is another natural material that can be used to synthesize hydrogels. It can cross-link under the action of divalent calcium ions, but the mechanical properties of the hydrogels produced are insufficient. Kalaf et al. had made changes to the cross-linking method, and generated a hydrogel with stronger mechanical properties and hydration, and a lower degradation rate (Growney Kalaf et al., 2017). In recent studies, alginate, like other natural polymers, has often been used as a components in composite hydrogels. For example, Sun, Z et al. used alginate as a hydrogel scaffold to load perfluorotributyl amine (PFTBA) as an oxygen regulator to treat IVDD (Sun Z. et al., 2016). Panebianco et al. used oxidized alginate to make microspheres and genipin-crosslinked fibrin to form a hydrogel system to deliver AF cells for IVDD treatment (Panebianco et al., 2022). The authors designed a composite material termed FibGen + MB-RGD that can effectively carry microencapsulated AF cells and eliminate the cytotoxicity of genipin. This material has high biomechanical stability and promotes ECM synthesis. Animal experiments have also proven that this material can restore the mechanical characteristics of the IVD after injury, prevent further IVDD, and promote ECM regeneration in the defect space.

Hydrogels made of a single natural polymer often have certain performance defects. For example, alginate hydrogels exhibit desirable porosity and hydrophilicity but poor cell affinity; pure fibrin hydrogels have good cell adhesion and biocompatibility but cannot achieve ideal mechanical strength for the treatment of intervertebral discs. Therefore, in the last 10 years, the abovementioned natural polymers have often been used as components in composite hydrogels, complementing the properties of other hydrogel components to improve drug delivery, cell delivery and other functions. In recent studies, these natural materials have often been modified (e.g., by methacrylation) to create new hydrogels or microspheres or have been combined with synthetic polymers, peptides, or other ingredients to create new materials with higher mechanical strength or environmental responsiveness. Basic materials with different characteristics, are chosen, modified, and combined to design specialized hydrogels with suitable properties for treating different diseases and different pathological environments and with the ability to deliver different therapeutic factors, such as drug molecules, proteins, and RNA. These specialized hydrogels will play important roles in the clinic.

#### 3.1.2 Synthetic polymers

Despite the unique advantages of natural hydrogels in terms of biocompatibility and their use as extracellular matrices, their weak mechanical structure, their susceptibility to degradation *in vivo* and the uncontrolled molecular weight of the polymers at the time of extraction limit their application in hydrogels. Therefore, there is a need for polymers with precisely controlled molecular weights and compositions that can be reproducibly manufactured to exhibit specific mechanical properties, appropriate gelation behavior, and optimized drug/cell loading in the context of different disease treatment requirements. Synthetic polymers have emerged and in recent studies a variety of polymeric materials have been developed such as polyethylene glycol (PEG), polyvinyl alcohol (PVA), and polyacrylates.

##### 3.1.2.1 PVA

PVA is a biodegradable semicrystalline synthetic polymer. It is often used in the pharmaceutical field to prepare solid dispersions to improve the solubility of drugs (Rivera-Hernández et al., 2021), and can also be made into granulating liquids to slow crystal growth (Gaaz et al., 2015). Because the mechanical structure of PVA is similar to that of cartilage, it can be used to make biomaterials to replace the nucleus pulposus. The earliest method of cross-linking PVA was through chemical cross-linking or freeze-drying, but chemical cross-linking often weakens the biocompatibility of hydrogels. The freeze-drying method leads to slow gelation, and polyphenol molecules are now often used to cross-link PVA into supramolecular hydrogels. Chen et al. designed a hydrogel with shape memory by using PVA to form multiple hydrogen bonds with TA (Chen et al., 2018). Esteban et al. induced PVA cross-linking through different small molecule polyphenols, and made thermally reversible hydrogels by exploiting the reversibility of hydrogen bonds (Euti et al., 2019). PVA hydrogels also provide controlled drug release, which is highly useful in monitoring the pharmacokinetics of the drugs carried (Chao et al., 2020). PVA, because of its desirable mechanical properties and controlled drug release, is not only a highly suitable alternative material for supporting the IVD but also an excellent long-term anti-inflammatory material for delivering drugs to degenerative IVDs.

In recently reported studies, PVA-containing hydrogels have typically been used to fabricate highly biocompatible materials to replace the NP and AF, allowing NPCs to proliferate within them (Binetti et al., 2014; Suryandaru et al., 2021; Jia et al., 2022a). Given the aforementioned characteristics of PVA, the use of PVA as a hydrogel component should be considered as a way to enhance the mechanical properties of hydrogels to better replace IVDs and provide slow drug release for the long-term treatment of IVDD.

##### 3.1.2.2 PEG

PEG hydrogels have been widely used in the past for their excellent drug-carrying and cell-transporting capabilities, as well as their excellent biocompatibility (Nuttelman et al., 2008). The hydrogel formed by PEG “click” chemistry provides versatile options for bioconjugation and can form stable covalent bonds under the action of copper ions, and the resulting hydrogel has excellent mechanical properties (Kolb et al., 2001). PEG can also form light-responsive gels with diacetylene-functionalized allyl ester peptides (Polizzotti et al., 2008). Moreover, PEG hydrogels have the property of repelling nonspecific protein adsorption and cell adhesion, which prevents inflammatory cells from adhering to the surface of the hydrogel: thus, PEG hydrogels carrying drugs or specific proteins can specifically enable the desired cells to grow within them without being affected by inflammation, giving such hydrogel an important role in the treatment of IVDD (Csucs et al., 2003; Lin and Anseth, 2009).

In the most recent studies, PEG containing hydrogels have been used to carry genes or drugs for the treatment of IVDD (Chen et al., 2020) or synthesized with natural materials to construct replacement tissues for IVD (Huang et al., 2023a). As indicated by reports published in other fields, PEG also has advantages in the fabrication of phase change materials (Ma et al., 2023) and sensors (Yuan et al., 2023). Improving this feature and designing environmentally responsive hydrogels based on the environmental characteristics of IVDD could lead to a wider range of uses for PEG in the treatment of IVDD.

##### 3.1.2.3 Polylactic-glycolic acid (PLGA)

PLGA is a hydrophobic polymer. The insertion of hydrophobic sequences such as PLGA into an otherwise hydrophilic hydrogel can achieve effects that are not possible with ordinary hydrogels, such as delivering hydrophobic drug molecules into the body (Makadia and Siegel, 2011). PLGA is widely used in the delivery of targeted drugs because of its unique biodegradability, adsorption, and high biocompatibility (Jin et al., 2021; Rahmani et al., 2023). Hydrogels containing PLGA have many applications in bone treatment. Glin et al. used PLGA-g-PEG to deliver hydroxyapatite (HAP) to treat bone defects (Lin et al., 2012), M. Zewail et al. used chitosan/β-GP hydrogels loaded with DEX/PLGA NPs and LEF/NLC hydrogel systems to promote joint healing (Zewail et al., 2021). J. Yang et al. utilized concentric ring arrangements of electrospun (ES) polycaprolactone (PCL)/poly (D,L-propylglycolide-ethylglycolide) (PLGA)/collagen compositions based on type I collagen(PPC) to make an AF replacement for IVDD treatment (Yang et al., 2017). The most commonly fabricated hydrogel containing PLGA is the temperature-responsive PLGA-PEG-PLGA hydrogel. This hydrogel has unique advantages in delivering anti-inflammatory drugs into the IVD to treat IVDD and can provide mechanical support and anti-inflammatory effects at the same time after entering the IVD (Yan et al., 2015).

PLGA is currently used mostly as a component of temperature-sensitive hydrogels, as a simple drug delivery carrier, and in the form of microspheres to achieve controlled drug release. There is little research on PLGA-containing hydrogels (Chung et al., 2016; Sayiner et al., 2020). In the future, PLGA hydrogels microspheres designed for their ability to carry hydrophobic drugs can also be chemically or physically modified to improve their temperature sensitivity and thus their better *in situ* gel-forming ability, enabling broader use in treating IVDD.

##### 3.1.2.4 Polyurethane (PU)

PU is composed of isocyanate and urethane groups polymerized with hydroxyl macromolecules as the basic repeating unit and has good abrasion resistance, toughness, and corrosion resistance (Liu Y. et al., 2021; Jiang R. et al., 2023), which are advantages in the production of biomaterials for replacing IVDs or AFs. J. Hu et al. developed a silk fibroin/polyurethane (SF/PU) hydrogel that is liquid or semiliquid prior to injection and undergose gelation *in situ* after injection into the intervertebral disc, forming a gel with outstanding mechanical properties as a prosthetic biomaterial for replacing the NP and enabling strong visualization on T2-weighted MRI images allowing it both to provide adequate mechanical support and can also be readily visualized during subsequent testing (Hu et al., 2012). In unconfined compression tests, the SF/PU hydrogel exhibited a nonlinear strain curve, showing a sharp increase in the compression modulus with increasing pressure. Rheological tests also revealed that the material possesses higher hardness and stronger elasticity than natural medullary tissue. Therefore, it can be used as a postsurgical disc filling material. In recent years, a small number of hydrogels made of PU have been used for the treatment of IVDD. However, composite hydrogels with PU as a component can have high mechanical strength and are suitable for replacing the AF and NP (Hu et al., 2012; Hu et al., 2017; Du et al., 2020). In other fields, composite hydrogels composed of PU are commonly used for drug delivery; accordingly, microspheres composed of PU can also be used for drug and cell delivery in the future treatment of IVDD, and hydrogels with large pores and high mechanical strength can be developed for the attachment and growth of NPCs (Hsieh and Hsu, 2019; Wang Q. et al., 2023).

In summary, although hydrogels consisting of artificially synthesized polymer materials have many advantages, such as stable and controllable molecular weight, mechanical properties that can be changed as needed, and variable affinity for carriers such as drugs, genes, and cells, their greatest disadvantage is difficult degradation, which limits their clinical application. Therefore, when designing future hydrogels containing synthetic polymers for IVDD therapy, it will be beneficial to modify them to form hydrogels or microspheres with strong mechanical properties and the ability to respond to environmental stimuli, which will be able to carry therapeutic factors such as drugs and cells and to undergo stimulus-responsive cleavage into small molecules to metabolize.

## 4 Stimuli-responsive hydrogels

In recent years, composite hydrogels designed to respond to specific stimuli to meet the needs of treating different diseases have attracted extensive attention due to their unique advantages. This type of hydrogel enters the lesion in a sol state, and can quickly form a gel or release loaded drugs under specific conditions such as light, temperature, pH and other stimuli. Stimuli are mainly categorized into physical stimulation, and chemical stimulation. Stimuli-responsive hydrogels have been widely used in drug delivery, gene delivery, cell delivery and other fields. For the treatment of IVDD, hydrogels should satisfy the following conditions: 1) they can provide sufficient mechanical support, 2) they are injectable, 3) they have a long-lasting anti-inflammatory effect and 4) they exhibit on-demand smart drug release. Therefore stimuli-responsive hydrogels have a wide range of applications in the treatment of IVDD.

### 4.1 Inflammation-responsive hydrogels (ROS and pH)

#### 4.1.1 Inflammatory mediators as the basis of the response

Compared with the normal IVD, IVDD provides an inflammatory microenvironment characterized by local acidification and ROS accumulation. Hence, pH and ROS are natural triggers for drug delivery from smart hydrogels at inflammatory IVDD sites, and inflammation (pH/ROS)-responsive hydrogels may be one of the best candidates for IVDD regeneration. pH-responsive hydrogels are the most basic inflammation-responsive hydrogels, and the swelling process of such hydrogels usually relies on a change in the charge of the matrix (Ding et al., 2022). The mechanism of the response to inflammation is usually the ionization of polymer sidechains and the generation of electrostatic repulsive forces within the polymer network that cause the hydrogel to swell or shrink, and the degree of swelling changes with the pH (Oh et al., 2009). Alternatively, the response to inflammation can be achieved by inverse bonding. In an inflammatory environment, which is often accompanied by a decrease in the pH of the surrounding matrix, the pH-sensitive ligand bonds in the hydrogel will break resulting in rapid hydrogel liquefaction to achieve the release of the drug specifically in the inflammatory environment. Research by numerous researchers has produced, new inflammation-responsive hydrogels with strong sensitivity to pH and ROS. Hydrogels can be endowed with a wide range of functionalities by Schiff base bonding, borate bonding, disulfide bonding, etc., opening up possibilities for clinical applications.

#### 4.1.2 Hydrogel design concept

**FIGURE 3:**
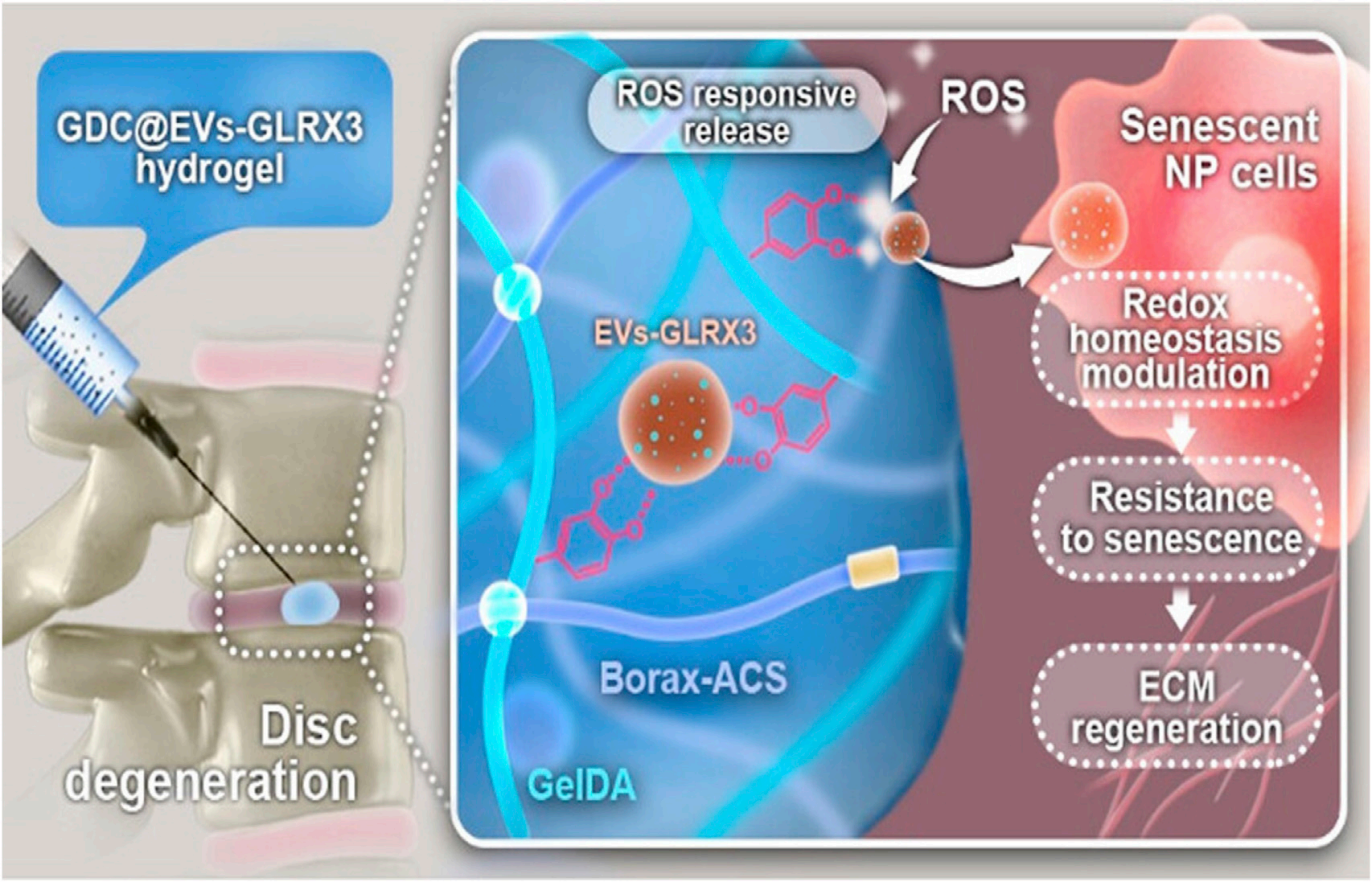
Inhibition of ROS and regulation of redox homeostasis using ROS-responsive GDC@EVs-GLRX3 hydrogel delivery system for the treatment of IVDD (Liu et al., 2023)

Inflammation-response hydrogels are being updated with newer iterations (Hassanzadeh et al., 2019). Zhang et al. used polyacrylic acid (PAA) as the basis for a porous network of polymer hydrogels modified by chemical cross-linking with N-hydroxysuccinimide (NHS) and HA, which is useful in the field of wound healing (Zhang X. et al., 2022). Wang, Y et al. utilized the ability of phenylboronic acid (BA) to form reversible boron ester bonds with diol-carrying molecules, which exhibit pH and ROS sensitivity, to design new inflammation-responsive hydrogels by grafting BA onto gel and then grafting β-cyclodextrin (CD) and tannic acid (TA) onto the backbone, to deliver curcumin with miRNAs for the treatment of IVDD through a response to the pH in the degenerative IVD. Curcumin is released on demand in the IVD in response to pH for its anti-inflammatory effect, and the sustained release of the carried miRNA also promotes the regeneration of the ECM, which is very helpful for the treatment of IVDD (Wang Y. et al., 2022). With the gradual advancement of inflammation-responsive hydrogel research, ROS-responsive hydrogels have also been developed. C Liu et al. prepared inflammation-responsive hydrogels with ROS responsiveness by cross-linking borax-coupled aldehyde-modified chondroitin sulfate (Borax-ACS) with the ECM-derived component dopamine (DA)-functionalized gelatin (GelDA) carrying ROS-based glutaredoxin3 (GLRX3), thereby developing GLRX3+ mesenchymal stem cell-derived extracellular vehicles (EVs-GLRX3), which treated IVDD by enhancing the cell antioxidant capacity. A diagram of the mechanism can be seen in Figure 3 (Liu et al., 2023). In addition to their drug-carrying function, hydrogels can perform various physiological functions through the grafting of additional chemical moieties. Q Zheng et al., synthesized the ROS-responsive polymer methoxy poly(ethylene glycol)-b-poly(propylene sulfide) (mPEG20-b-PPS30) carrying the peptide MR409, and when the resulting ROS-responsive hydrogel was injected into degenerative IVDs, it depleted some of the ROS and simultaneously released MR409 for anti-inflammatory purposes, which was shown to be effective in the treatment of IVDD (Zheng et al., 2021). Therefore, inflammation-responsive hydrogels are promising for application in IVDD, where long-term inflammation exists in degenerative IVDs, and long-term anti-inflammatory effects are achieved by the slow-release of drugs in response to pH.

#### 4.1.3 Trends in research

In addition to medication, many biomaterials can be delivered by hydrogels; for example, microspheres can encapsulate cells, genes, and drugs to assist the regeneration of the ECM while exerting anti-inflammatory effects to promote the recovery of IVDs. Therefore, inflammation-responsive multiscale-based delivery systems have been developed as a promising strategy for the treatment of IVDD. However, more research is still needed on how to maintain a high loading rate, inflammatory responsiveness, and high biocompatibility at the same time.

### 4.2 Temperature sensitive hydrogels

#### 4.2.1 Temperature sensitive hydrogel mechanisms

Temperature-responsive hydrogels undergo a phase transition at a critical temperature, which is often referred to as the lower critical solution temperature (LCST) or critical solution temperature (UCST). A common feature of temperature-sensitive hydrogels is the presence of hydrophobic groups, and there are two gel-forming processes, namely, sol-shrinkage and sol-gel transition. Most temperature-sensitive hydrogels are less water-soluble at elevated temperatures, and the polymer molecules shrink to form hydrogels at temperatures above the LCST. The polymer chains of such hydrogels usually contain a moderate amount of hydrophobic groups or a combination of hydrophilic and hydrophobic groups. At low temperatures, hydrogen bonds formed between the hydrophilic molecules of the polymer chain and water molecules play a dominant role, so that the polymer solubility in water is higher. With increasing temperature, the role of hydrogen bonding is gradually weakened, and the interaction between the hydrophobic groups is enhanced, so the polymer undergoes shrinkage to form a hydrogel. This category is represented by poly(N-iso-propylacrylamide) (PNIPAAm). When the polymer chain is noncovalently cross-linked, a sol-gel transition occurs at the appropriate temperature, and the sol form occurs at higher temperatures. This category is represented by poly (ethylene oxide) (PEO) and poly (propylene oxide) (PPO) block copolymers forming PEO-PPO-PEO.The temperature-responsive hydrogels commonly used in IVDD preparation for drug delivery are hydrogels with an LCST (Suntornnond et al., 2016). Because this type of hydrogel is hydrophilic at temperatures lower than the critical temperature, it is liquid, which makes the hydrogel injectable. If the human body’s physiological temperature is higher than the critical temperature of the hydrogel, then when the hydrogels is injected into the IVD, the hydrophobicity of the hydrogel increases, and the hydrogel begins to “solidify”, providing mechanical support for the degrading IVD.

#### 4.2.2 Temperature sensitive hydrogels in the treatment of IVDD

The most common temperature-responsive hydrogel used in recent years is the PLGA-PEG-PLGA (PPP) triblock copolymer hydrogel, which has been reported to be used to treat IVDD, e.g., Chen, Q, et al. used an injectable PPP hydrogel to deliver bevacizumab, a vascular endothelial growth factor (VEGF) antagonist, for the treatment of IVDD (Chen et al., 2022). Pan et al. synthesized an aminated hyaluronic acid-g-poly(N-isopropylacrylamide)-gefitinib (AHA-g-PNIPAAm-gefitinib) hydrogel to deliver gefitinib for the treatment of IVDD (Pan et al., 2018). Both of the abovementioned drug-carrying temperature-responsive hydrogels showed the superiority of the temperature-responsive hydrogel for IVDD treatment. In addition, there are studies on the use of temperature-responsive hydrogels as an alternative to IVDs. Li, Z et al. synthesized a thermosensitive hydrogel with enhanced stabilization by the N-acetylation of glycol chitosan (GC), and the critical temperature could be adjusted according to the degree of acetylation and the concentration of the polymer within the range of 23°C–56°C. The results of the experiments demonstrated that this hydrogel has good biocompatibility and is able to provide adequate mechanical support for degenerative IVD sites in experimental animals and to maintain stability for a long period of time (Li et al., 2018). PNIPAAm is a material widely used in the design of temperature-sensitive hydrogels. C. Sammon and Prof. C.L. Le Maitre investigated a novel pNIPAM-DMAc-Laponite hydrogel system based on PNIPAAm. The system has excellent thermosensitivity and injectability and is able to promote the differentiation of MSCs into the NPC phenotype in a hypoxic environment without the need for additional differentiation factors. In subsequent experiments, the authors demonstrated that the ECM of MSCs cocultured with this hydrogel system showed significant increase in the content of type II collagen and chondroitin sulfate, as well as a similar increase in NPC markers, indicating that the system provides an effective and convenient strategy for the repair of IVDD (Thorpe et al., 2016).

**FIGURE 4:**
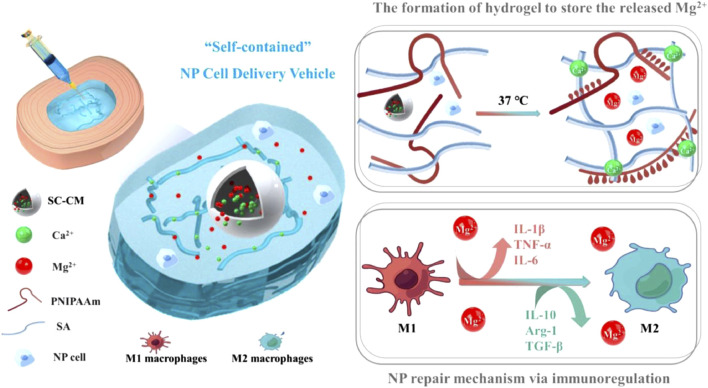
Schematic showing the composition of the injectable self-contained SA/PNIPAAm hydrogel, its working principle and functionality (Jiang Y. et al., 2023)

**FIGURE 5:**
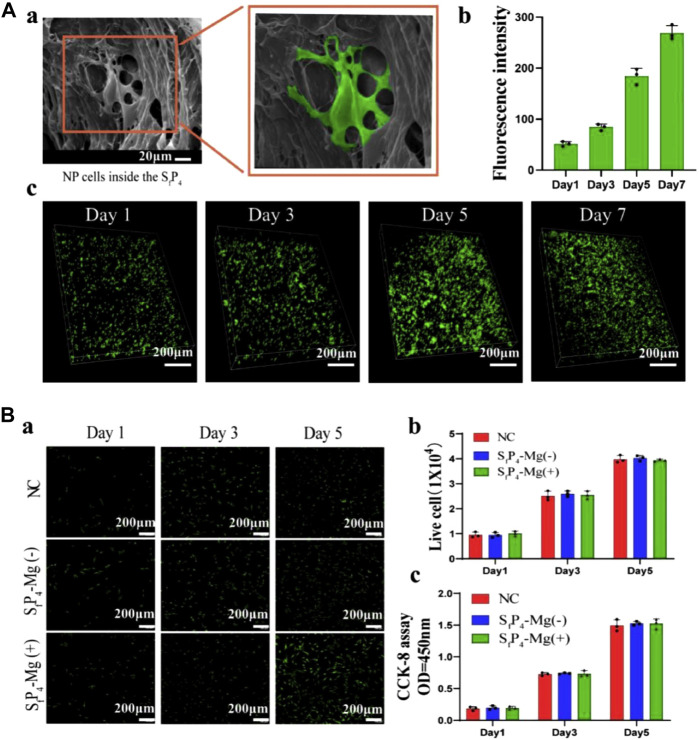
**(A) (a)** SEM of NP laden SfP4 hydrogel on day 5; **(b)** Live-cell counting and **(c)** 3D live/dead assays of NP laden SfP4 on day 1,3,5,7. **(B) (a)** The live/dead assays; **(b)** The live-cell counting; **(c)** The CCK-8 assays of NPCs on day 1,3,5 co-cultured with different hydrogels. (Scale bar, 200 µm) (Jiang Y. et al., 2023)

**FIGURE 6:**
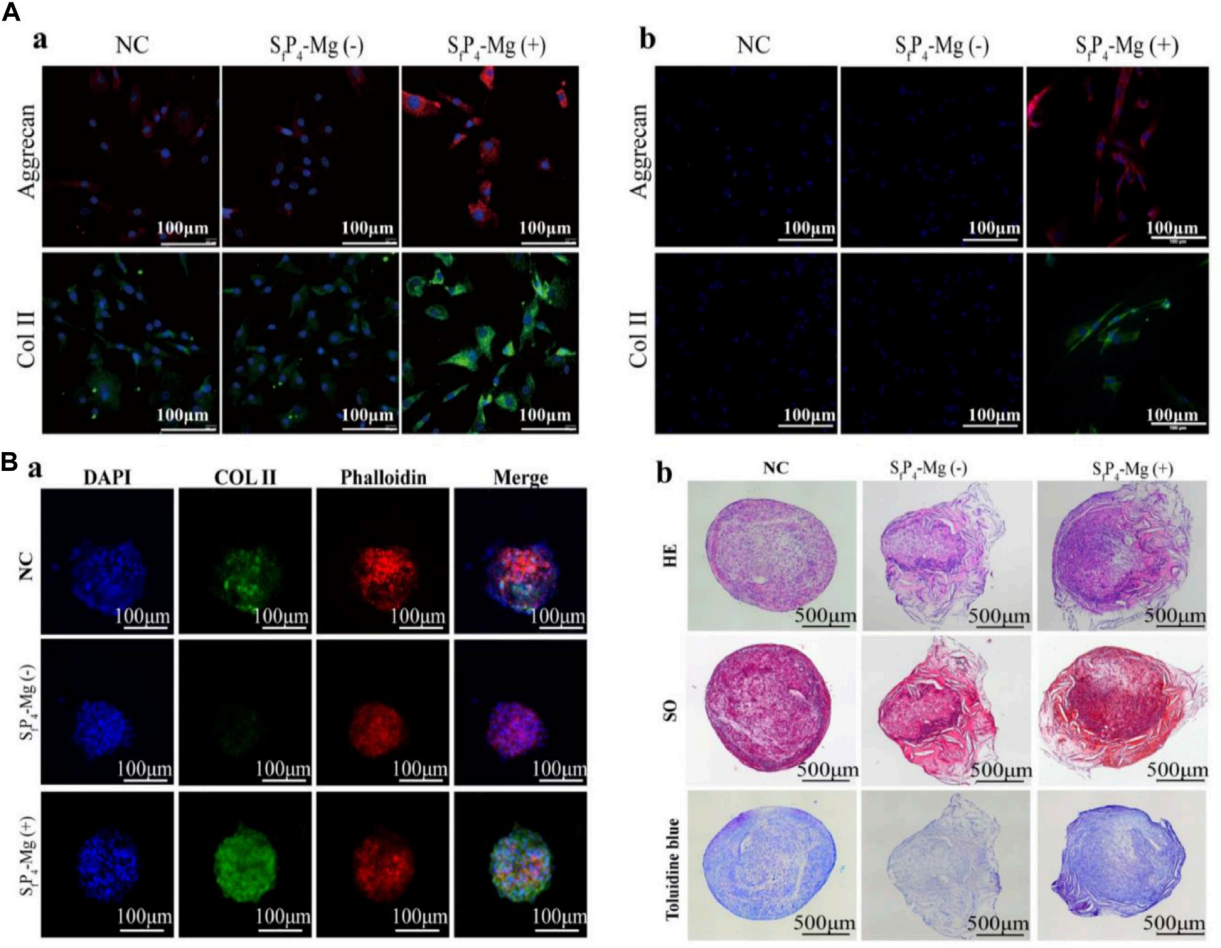
**(A)**. Col II, Aggrecan expression and actin cytoskeleton of **(a)** NPCs and **(b)** BMSCs in different hydrogels detected by the immunofluorescence. **(B) (a)** Immunofluorescence staining for Col II (green), actin cytoskeleton (red), and cell nuclei (blue) of BMSCs 3D pellet cultures in different hydrogel; **(b)** H&E, Safranin O and Toluidine Blue staining of BMSCs 3D pellet sections (Jiang Y. et al., 2023)

**FIGURE 7:**
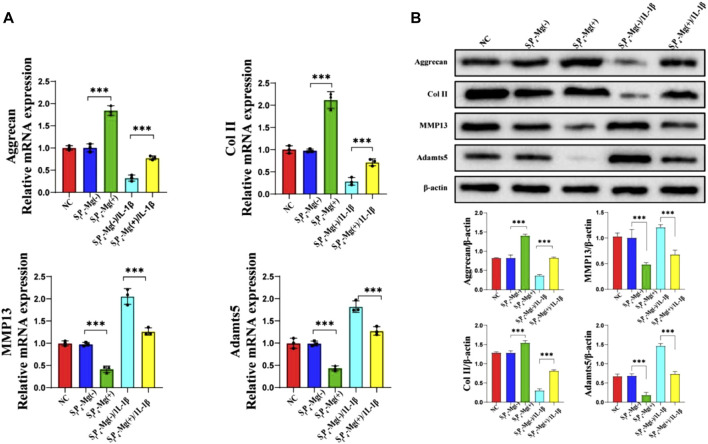
**(A)** qRT-PCR; **(B)**. Western bolts results of aggrecan, COL II, MMP13 and Adamts5 expressions for NPCs encapsulated in S_f_P_4_-Mg (+), S_f_P_4_-Mg (−), S_f_P_4_-Mg (+)/IL-1β and S_f_P_4_-Mg (−)/IL-1β hydrogels (***p < 0.001) (Jiang Y. et al., 2023)

In a recently reported paper, Y. Jiang et al. investigated a hydrogel delivery system consisting of sodium alginate (SA), poly(N-isopropylacrylamide) (pNIPAAm), and silicate ceramics (SC), carrying NPCs and Mg^2+^ (Jiang Y. et al., 2023). At human body temperature, pNIPAAm and SA form interpenetrating network (IPN) hydrogels *in situ* in the presence of temperature and Ca^2+^ ions and encapsulate NPCs and Mg^2+^ (Figure 4). As shown in Figure 5, the hydrogel possesses excellent biocompatibility. Next, to determine the effect of Mg^2+^ on NPCs, two types of hydrogels containing Mg^2+^ and without Mg^2+^ were prepared, and as shown in Figure 5B, the presence of Mg^2+^ did not affect the proliferation of NPCs. The authors further explored the regenerative effect of NP ECM by coculturing NPCs and BMSCs loaded into hydrogels, and the increase in Col II and aggrecan promoted by hydrogels in the presence of Mg^2+^ can be seen in Figure 6. The results of 3D pellet culture and Safranin O and Toluidine Blue staining demonstrated that hydrogels carrying Mg^2+^ can effectively promote the synthesis of NP cellular stroma to achieve the repair of degenerative IVDs. It is worth mentioning that for the repair of degenerative IVDs, in addition to the need to promote the regeneration of the ECM, the anti-inflammatory effect is a point worth evaluating. In this study, the authors used IL-1β to mimic the inflammatory situation within NPCs, and as seen in Figure 7, the Mg^2+^-carrying hydrogel group was able to attenuate the inflammatory damage to the ECM and justify the above conclusion of promoting ECM regeneration. The hydrogel system delivers NPCs with Mg^2+^ into the degenerative NP for repair, which confirms that the system can effectively delay the degeneration of IVDs, and based on the ability of the hydrogel to form an IPN network under the action of temperature and Ca^2+^, it can provide excellent injectability and mechanical properties at the same time, which is an advance for therapeutic strategies for IVDD.

#### 4.2.3 Trends in research

Temperature-responsive hydrogels have great advantages in the treatment of IVDD due to their injectability, *in-situ* gelation ability, and rapid gelation when the LCST is reached. They can enter the IVD in a minimally invasive way to avoid secondary damage, while *in situ* gelation prevents leakage and provides mechanical support for the degenerative IVD. These properties provide great application prospects for the treatment of IVDD.

### 4.3 Enzyme-responsive hydrogels

#### 4.3.1 Enzyme response mechanisms

Enzyme-responsive hydrogels typically contain amino acid side chains that can be covalently linked by enzymes to form peptides. The peptides can deform or degrade the structure of the hydrogel when catalyzed by specific enzymes. The hydrolysis products of peptides are amino acids; therefore, enzyme-responsive hydrogels containing peptides and their derivatives are widely used because of their high biocompatibility, low immunoreactivity, controlled degradation time, and potential as slow-release carriers (Gao et al., 2022; Wu et al., 2022).

#### 4.3.2 Enzyme-responsive hydrogels in the treatment of IVD

**FIGURE 8:**
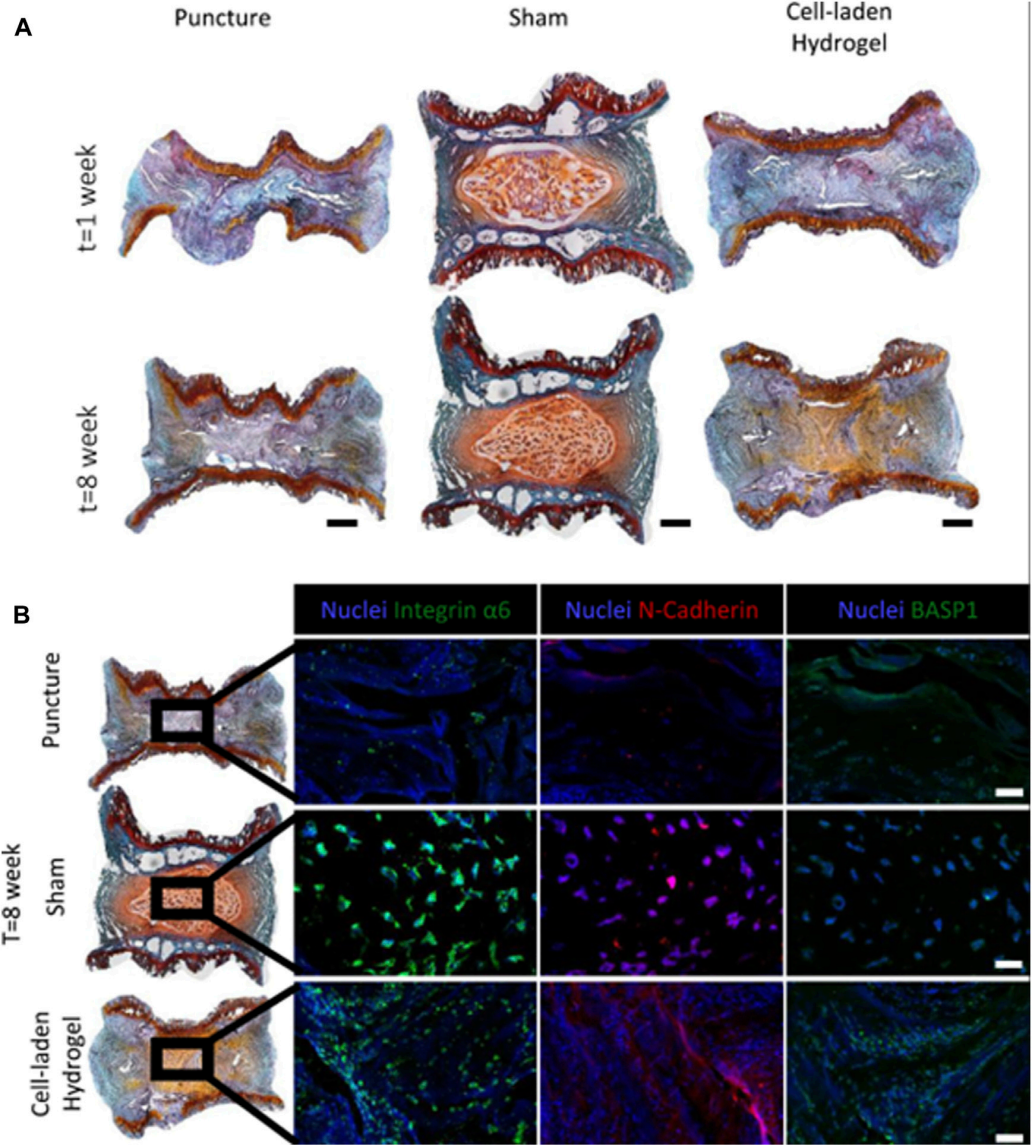
Application of enzyme responsive hydrogel in IVDD, histological assessments of the IVDs. **(A)** 20 μm thick tissue sections, stained via SafO/Fast Green/Haematoxylin. Scale bar is 500 μm. **(B)** At the eighth week post-injury timepoint, differences in protein expression levels between groups was apparent. Although protein expression in the cell-laden hydrogel condition appeared to resemble that of the naïve condition, morphological differences in cell distribution could still be observed. Scale bar is 250 μm (Barcellona et al., 2021)

The earliest enzyme-responsive hydrogels were synthesized by Yui et al. from polyethylene glycol and deoxyglucose by sequential cross-linking reactions, and *in vitro* degradation was achieved by papain and dextranase (Kurisawa et al., 1997). The use of this type of hydrogel in the repair of IVDs has been reported. Matrix metalloproteinase (MMP) is a common protease with detrimental effects in IVDs (Vo et al., 2013), as it contributes to the degradation of the ECM; thus enzyme-responsive hydrogels used to treat IVDD are usually designed to target MMP. A pH- and enzyme-responsive hydrogel called Col-JK1 was designed by Zheng et al. In degenerative IVDs, the acidic environment and high expression of MMP caused the hydrogel to degrade rapidly and release hydrogen sulfide (H_2_S), which inhibited the degeneration of myeloid cells and modulated the NF-κB signaling pathway to inhibit inflammation (Zheng et al., 2019). Marcos N et al. used PEG with laminin (LM) and the peptides IKVAV and AG73 to create a novel hydrogel that not only provided sufficient mechanical support for degenerative IVDs but also restored the juvenile myeloid phenotype to degenerative human myeloid cells, as seen in Figure 8. This hydrogel was able to increase the height of the injected IVD after 8 weeks, and the staining results were also able to show that the hydrogel loaded with cells resulted in protein expression in the NPs similar to that of the juvenile phenotype, which indicated effective repair of the degenerative IVDs. (Barcellona et al., 2020; Barcellona et al., 2021). Brian M et al. designed an NHPH system consisting of a self-assembled peptide hydrogel, MnO_2_, and growth differentiation factor-5 (GDF-5). The entire NHPH system has mechanical strength similar to that of IVDs, inhibits inflammation, and delivers slow-release growth factors that enhance chondrocyte differentiation, thereby promoting the regeneration of fibrocartilage tissue for the treatment of degenerative IVDs (Conley et al., 2023). In summary, the excellent and controllable specific degradation of enzyme-responsive hydrogels, as well as their effects on cellular behavior, can provide new solutions for the treatment of IVDs (Feng et al., 2018).

#### 4.3.3 Constraints and challenges

Enzyme-responsive hydrogels have been widely studied for their special properties of controlled drug release even under mild conditions. However, enzyme-responsive hydrogels must fulfill a number of criteria (Yan et al., 2021). First, a sufficient grafting rate of the enzyme reaction substrate in the hydrogel network needs to be achieved to accomplish the enzymatic reaction. Morevoer, the enzymatic reaction must cause significant changes in the hydrogel structure to accomplish drug release. Due to these limitations, enzyme-responsive hydrogel drug delivery systems are still in development. Developing efficient enzyme-responsive hydrogel drug delivery systems with better biocompatibility and degradability will be a challenge for future researchers.

### 4.4 Photosensitive hydrogel

#### 4.4.1 Photosensitive hydrogel mechanisms

Photosensitive hydrogels have natural advantages as a tools for drug delivery. Compared to pH-sensitive hydrogels, whose sensitivity is limited by the migration rate of H^+^, and temperature-sensitive hydrogels, whose sensitivity is limited by the efficiency of thermal conductivity, photosensitive hydrogels allow easier and more rapid control. A common feature of photoresponsive hydrogels is the presence of photosensitive functional groups [e.g., (O-nitrobenzyl)] (Qiu and Park, 2001). The covalent linkage between the photosensitizing functional groups and the main chain of the hydrogel polymer is a key factor in the swelling and shrinkage of photoresponsive hydrogels without separation. This type of hydrogel has been widely used in tissue engineering. Common photo-sensitive hydrogels are categorized as UV-sensitive and visible light-sensitive hydrogels, and the photosensitive hydrogels reported in the literature for IVDD treatment are often UV-sensitive hydrogels. In 1989, A Mamada et al. introduced a leuco derivative molecule, bis(4-(dimethylamino)phenyl) (4-vinylphenyl)methyl leucocyanide, into the polymer network, In the first observation of the phototropic phase transition process in gels (Mamada et al., 1990), triphenylmethane derivatives dissociated under UV irradiation to produce triphenylmethyl cations, and as the number of ion pairs increased, the sol started to swell.

#### 4.4.2 Photosensitive hydrogels in the treatment of IVDD

**FIGURE 9:**
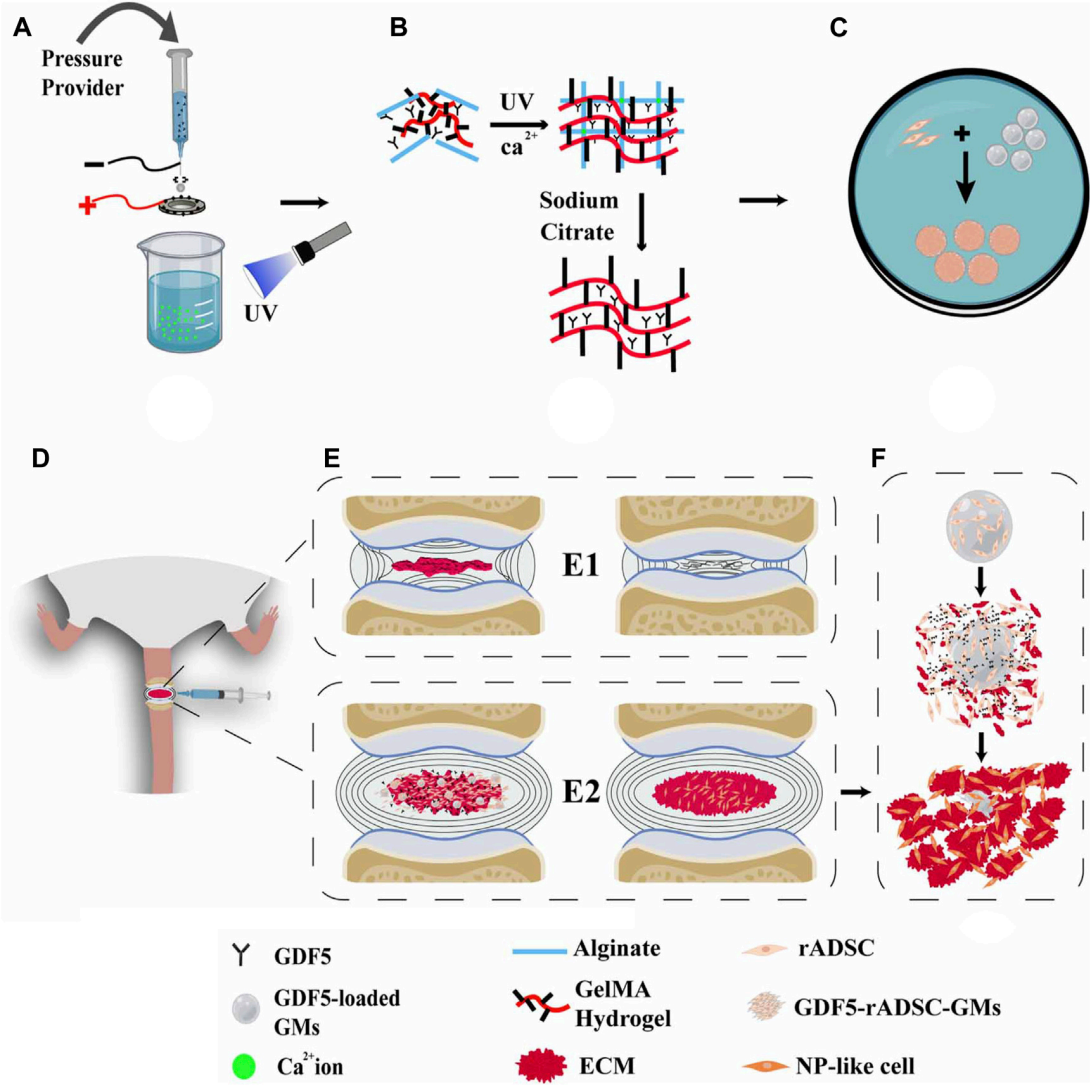
**(A)** Diagram of the electro-assisted printing device for the preparation of GDF5-GMs. **(B)** Schematic diagram of GDF5-loaded GM formation. **(C)** rADSCs were cultivated on the microspheres. **(D)** Construction of DDD model mice and injection of GDF5-loaded GMs with rADSCs into the degenerative intervertebral disc. **(E)** (E1), (E2) The progress of DDD in degeneration and repair groups. **(F)** Coordination mechanism of the cells–GFs–GMs system (Xu et al., 2020).

GelMA is a photoresponsive hydrogel currently receiving particular attention, and its precursor substance, gelatin, is a denatured collagen with lower antigenicity. Gelatin is modified by methacryloyl to obtain GelMA (Yue et al., 2015; Klotz et al., 2016; Wang et al., 2018), which can be rapidly polymerized to form a gel under the action of ultraviolet light or photocuring agents and which is injectable and has controllable mechanical properties. Li et al. designed an injectable photocrosslinkable porous GelMA/serine protein glycidyl methacrylate (SilMA) hydrogel, in which MSCs were encapsulated, and they demonstrated that this hydrogel system could promote the M2 polarization of macrophages for anti-inflammatory treatment while delivering MSCs to promote cartilage repair (Li et al., 2023a). Theoretically, this hydrogel could also be applied in the treatment of IVDD. To enhance the anti-inflammatory effect of the hydrogel system, it is also possible to encapsulate drugs in GelMA, such as curcumin (Li X. et al., 2023). In recent studies, GelMA has been modified to carry drugs and cells for the treatment of IVDD. Y, Zhang et al. designed a porous HA-PEG/NAGA-GelMA hydrogel, with multiple hydrogen bonds, which endowed the hydrogel with excellent mechanical properties and was able to withstand the pressure of AF when performing AF repair (Zhang C. Y. et al., 2022). Y, Li et al. prepared fucoidan-functionalized gelatin methacryloyl microsphere(Fu@GelMA-MS) hydrogel microspheres, which were able to inhibit inflammation in the environment of IVDD and to reduce the inflammation of IVDD by activating nuclear factor erythroid 2-related factor (NRF2), inhibiting intracellular ROS and enhancing antioxidant enzymes to protect the ECM. The authors used a microfluidic device to integrate fucoidan into GelMA hydrogel microspheres to form the Fu@GelMA hydrogel system. The system was used to culture NPCs, and the results demonstrated excellent ability to promote cell proliferation with high biocompatibility, and fucoidan sustained release for 38 days, showing excellent controlled drug release. In a follow-up study, fucoidan was found to increase the gene expression and protein expression of three ECM-related genes, Aggrecan, Col II, and SOX9, demonstrating that fucoidan has a good ECM regeneration effect on NPCs. Simultaneous coculture of NPCs with Fu@GelMA in an inflammatory environment induced by IL-1β, showed that the system could resist ROS and increase the gene and protein expression of antioxidant enzymes (Nrf2, Ho-1, Sod1, etc.). In NPCs, the IVDD was successfully treated by combining anti-inflammatory effects and the promotion of ECM regeneration (Li et al., 2023c). H Xu et al. used GelMA and alginate to make photosensitive hydrogel microspheres carrying GDF-5 for the anti-inflammatory treatment of postoperative intervertebral discs. The mechanism diagram is shown in Figure 9. The drug-loaded microspheres were able to effectively stimulate the regeneration of ECM by NPCs (Xu et al., 2020). Thus, the light-responsive hydrogel is expected to be able to repair degenerative IVDs at multiple levels (AF replacement, anti-inflammatory, etc.) and can provide new ideas for the treatment of IVDD.

#### 4.4.3 Constraints and challenges

A main to be addressed in the future is how to develop photosensitive hydrogels that can respond to long wavelengths of light and energize the reaction with higher quantum efficiency. In the treatment of IVDD, the greater challenge is to develop photoreversible hydrogels. Most of the photosensitive hydrogels reported thus far have been photogelated *in vitro* and then injected into the IVD, and injectability is obtained at the expense of the mechanical properties of the hydrogel. Photosensitive hydrogels with photoreversibility will have a wider range of applications. Another issue that needs to be addressed is to improve the biocompatibility of photosensitive hydrogels, and a more in-depth evaluation of the intermediates and final products of the photothermal reaction, as well as its effects on proteins and genes, is needed.

### 4.5 Other stimuli-responsive hydrogels

#### 4.5.1 Electro-sensitive hydrogel

Electrosensitive hydrogels are usually composed of polyelectrolytes, which are capable of contraction or expansion in the presence of an electric field. Therefore, by controlling parameters such as the current intensity, the time of action, and the direction of the electric field, it is possible to realize the control of local deformation of hydrogels at the anode and cathode (Tanaka et al., 1982; Garland et al., 2011). Hydrogel deformation is often used in the manufacture of artificial muscles (Fitzgerald et al., 2015) and has been used for controlled drug release. X Ying et al. designed electrosensitive hydrogel nanoparticles (ERHNPs) modified with angiopep-2 (ANG) and loaded with the antiepileptic drug phenytoin sodium (PHT). The ANG-PHT-ERHNP complex can easily transport the drug into the brain, and fast release can be achieved by the application of an electric field, decreasing the severity of seizure onset (Ying et al., 2014). To date, no electrically responsive hydrogels have been applied to the treatment of IVDD, but this stimuli-responsive hydrogel also has good therapeutic prospects for IVDD and can be combined with electrical stimulation for the repair of spinal cord injuries.

#### 4.5.2 Pressure-responsive hydrogel

The idea that hydrogels may undergo pressure-induced phase transitions originates from thermodynamic calculations for uncharged hydrogels, which indicated that certain hydrogels can collapse at low pressures and swell at high pressures (Qiu and Park, 2012). This type of hydrogel is commonly used to fabricate heat-pressure sensors: for example, X Fu et al. proposed a stretchable and self-powered temperature‒pressure dual sensing i-skin based on thermogalvanic hydrogels (TGHs), which is helpful for fabricating the skin of intelligent robots (Fu et al., 2022). This type of hydrogel has not been used for the treatment of IVDD and is not applicable to the treatment of IVDD according to the literature reported thus far.

As is apparent from the above description of electrosensitive hydrogels as well as pressure-responsive hydrogels, electrosensitive hydrogels are more promising in the treatment of IVDD. The hydrogel has good injectability when not electrically stimulated, and when successfully introduced into the IVD, the hydrogel can be made to have sufficient mechanical properties by electrical stimulation to provide a space for the cells to grow and can release the therapeutic factors it carries for further treatment of the IVDD. In subsequent studies, the potential of pressure-responsive hydrogels in combination with other materials (e.g., microspheres) can be considered, as well as the consideration of other injectable sites that are pressure-responsive hydrogels with more controllable drug sustained-release properties.

## 5 Summary

In this review, we describe the development of materials for preparing hydrogels, from natural materials to synthetic polymer materials, focusing on the key role of stimuli-responsive hydrogels synthesized from different materials in IVDD treatment. IVDD is a disease caused by multiple factors, such as genetics, environment, and aging, but the full etiology is still unclear. Long-term inflammation and ECM degradation in IVDs are currently considered to be the key factors leading to IVDD. Hydrogel is a substance similar to ECM. After the onset of IVDD, the IVD is reduced in height and insufficiently supported. A suitable hydrogel can provide mechanical support for the degenerative IVD while guiding the regeneration of medullary tissue, and hydrogel-carried medications or stem cells can further promote the recovery of the IVD. Different types of stimuli-responsive hydrogels have excellent effects in the treatment of IVDD, although pressure-responsive hydrogels and electroresponsive hydrogels are not described in detail here. In the future, the design of hydrogels may be improved by a better understanding of the etiology and pathology of IVDD and by combining the latest materials and engineering technologies to enhance the repair of degenerative IVDs.
